# Standing on the shoulders of giants: young aphids piggyback on adults when searching for a host plant

**DOI:** 10.1186/s12983-018-0292-7

**Published:** 2018-12-06

**Authors:** Moshe Gish, Moshe Inbar

**Affiliations:** 1 Department of Natural Resources and Environmental Management, University of Haifa, Haifa, Israel; 2 Department of Evolutionary & Environmental Biology, University of Haifa, Haifa, Israel

**Keywords:** Hitchhiking, Host location, Parent-offspring conflict, Parental care, Phoresy

## Abstract

**Background:**

Upon the detection of imminent peril, pea aphids (*Acyrthosiphon pisum*) often drop off their host plant. Dropping in response to insect enemies is intermittent in nature, but when a mammalian herbivore feeds on their host plant, a large mixed-age group of aphids usually drops off the plant at once. Aphids that reach the ground are confronted with new, hostile environmental conditions and must therefore quickly walk toward a suitable host plant. The longer it takes an aphid to reach a host plant, the more it is exposed to the risks of starvation, desiccation and predation.

**Results:**

We found that young nymphs, which have limited mobility and high mortality on the ground, quickly climb on conspecific (not necessarily parental) adults and cling to them before the latter start walking in search of a plant. This “riding” behavior is likely to be adaptive for the nymphs, for it shortens their journey and the time they spend off a host plant. Adults however, seem to be irritated by the riding nymphs, as they often actively try to remove them.

**Conclusions:**

After dropping from the host plant, young aphid nymphs travel at least part of the way back to a plant on the backs of adults. For the riding behavior to take place, nymphs need to successfully find adults and withstand removal attempts.

**Electronic supplementary material:**

The online version of this article (10.1186/s12983-018-0292-7) contains supplementary material, which is available to authorized users.

## Background

**Fig. 1:**
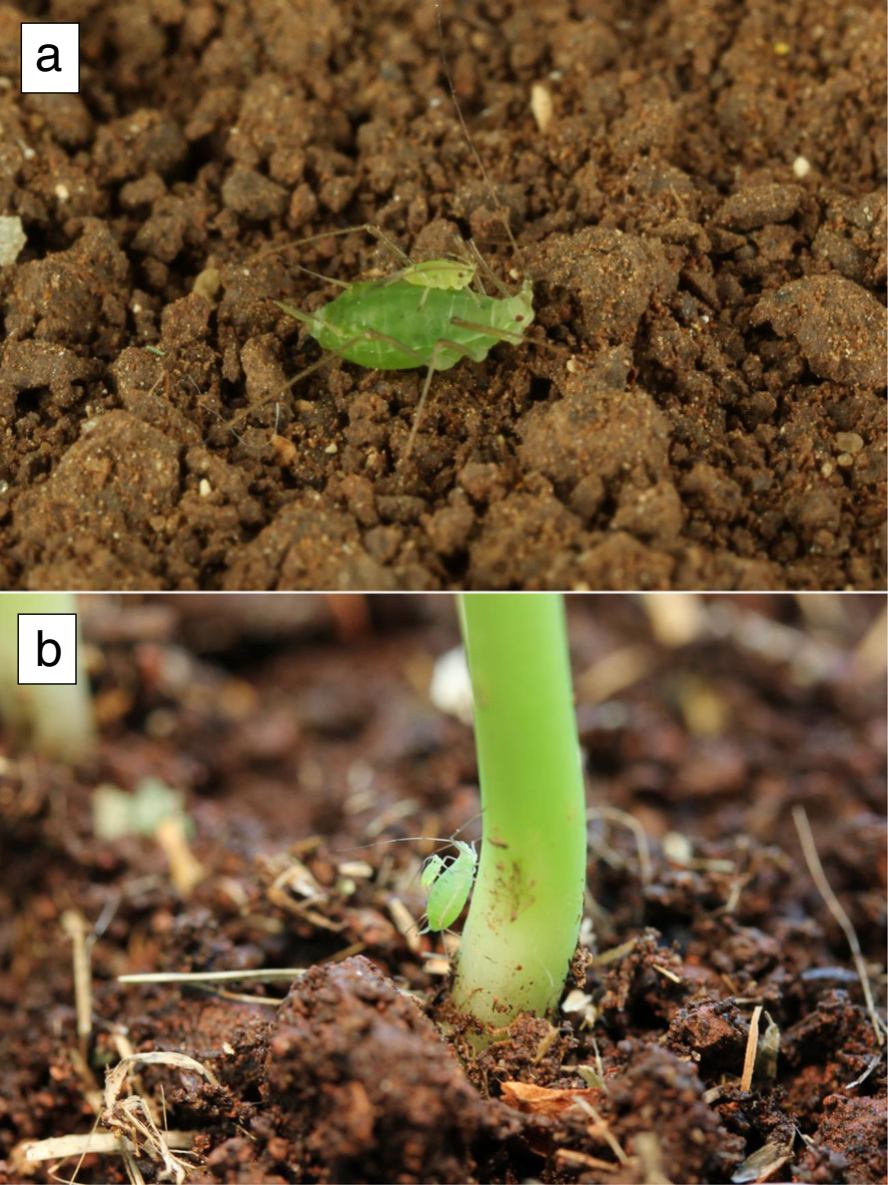
**a** A typical riding behavior of a fist-instar pea aphid nymph on an adult, after dropping off the host plant. **b** A nymph reaching a host plant on the back of an adult

Aphids (Hemiptera: Aphididae) live in colonies on plants, where they feed on phloem sap. On the plant, aphids are often attacked by predators and parasitoids [1]. Among aphids’ defenses against their natural enemies, behavioral defenses include walking away and dropping off the plant [2]. Some of the aphids that escape by dropping cling to plant parts they encounter on their way down, but those that do not, usually reach the surface [3, 4]. Dropping is an efficient way of evading an approaching enemy, but it comes with a cost: an aphid that reaches the ground is challenged by the need to return to the original host plant or find a new one, before it starves to death, becomes desiccated or falls prey to ground-dwelling predators [5, 6]. Despite these risks, the pea aphid (*Acyrthosiphon pisum*) often drops when it senses danger and after reaching the ground, it can disperse by walking to distances of over 13 m in 7 h [7]. When members of an aphid colony drop off the plant to avoid insect enemies, they do so in an intermittent manner: as the enemy advances through the colony, some of the aphids that sense the enemy or the alarm pheromone secreted by other colony members individually drop in irregular intervals [8]. However, a different pattern of dropping is observed when the colony is threatened by a foraging mammalian herbivore. When pea aphids (and some other species) sense the warm and moist breath of a mammalian herbivore and the plant movements it creates when it starts feeding, they immediately drop in large numbers- up to 60–80% of both adults and nymphs in the colony [8–11]. This emergency escape dropping may lead to numerous aphids being on the ground, having to search and find a suitable host plant. Movement from a landing site toward a new host plant is usually done by walking, as during most of the growing season adult pea aphids are usually wingless [12, 13]. Young nymphs are much smaller than adults (Fig. 1) and thus have an inferior ability to move on soil and plant debris compared with adults, which leads to higher mortality on the ground [7, 14, 15]. Natural terrain typically contains a variety of significant obstacles that can slow down young nymphs, such as cracks, stones, clumps of soil and fallen leaves and twigs. These surface features are relatively easily handled by adults, which have much longer legs than nymphs.

Here we report a novel behavior that occurs among pea aphids that have fallen to the ground. After the mass dropping of a pea aphid colony in response to the threat of mammalian herbivory, the young nymphs (mostly first and second instar) quickly walk toward adults and climb on their backs, where they stay as the adults start walking on the surface toward a host plant (Fig. 1; Additional files 1 and 2). We initially described and quantified this new “piggybacking” behavior (henceforth “riding”) and then tested the hypothesis that riding creates an intergenerational conflict, i.e. the nymphs initiate it, thus improving their chances of reaching a host plant, while the adults try to avoid it as it is a burden for them.


**Additional file 2:** Video of a first instar pea aphid riding on the back of an adult. (MOV 16480 kb)


Using laboratory experiments in which aphid movement was tracked on the surfaces of different arenas, we addressed the following questions: (i) Does the nymphs’ riding behavior differ from pea aphids’ natural tendency to climb on objects while walking on the ground? (see [16, 17]) (ii) How does riding behavior benefit or impede nymphs and adults? (iii) Is the riding behavior a type of parental care, and does genetic similarity between adults and nymphs (which is very high within an aphid colony) affect its frequency and duration?

## Methods

### Plants and insects

A pea aphids colony, originating from a parthenogenetic female collected in Kiryat Tiv’on, northern Israel, was reared in the laboratory on fava bean (*Vicia faba*) plants potted in commercial planting soil, at 22-24 °C, 65 ± 5% RH and a photoperiod of 16:8 L:D. Fava bean plants were used for experimentation 2 weeks after sowing. Aphid colonies that were used for experimentation were established on fava bean plants individually grown in plastic cups. For the ease of handling, the experimental plants were stripped of all leaves, leaving only a bare stem and an apical bud. Only wingless aphids were used in this study. The evening before each experiment, approximately 10 adult aphids (8–12) were placed on each fava bean plant. On the next morning, each plant was ready for experimentation with an aphid “colony” composed of adults and first instar nymphs (henceforth nymphs).

Lentil plants (*Lens culinaris*, a highly attractive host for pea aphids), used as destination host plants for surface-moving aphids, were planted in pots in commercial planting soil and after approximately 2 weeks, transplanted into the plant-surrounded arena (described below). Every morning the lentil plants in the arena were trimmed to a height of 10 cm. We chose lentil plants because they can be planted very densely (forming a highly uniform target) and they perform well after being trimmed repeatedly. Every 5 days, the lentil plants were replaced with new ones.

### Setup and working procedures

The experiments were performed in a fluorescent-light lit and air-conditioned laboratory at 22-24 °C. All experiments were performed under a 300 W halogen flood light that was situated 75 cm above the center of the working table, illuminating and moderately warming it (surface temperature of the experimental arenas was ~ 28 °C; surface temperature was measured using Extech© 42545 infrared thermometer). The different experiments in this study were performed using several types of arenas, as described below. In all experiments, two observers sitting on opposite sides of each arena tracked the aphids’ movement and behavior.

Aphids were induced to drop by tilting the fava bean plant so that the stem was 10 cm above the center of each arena, and then slowly exhaling on the stem (see Gish et al. 2010) while gently tapping on its base with a finger. In response to this stimulus, the average ratio of the nymphs:adults that dropped from our created colonies to the surface at the start of the experiments was 2.8 ± 0.2 (6.5 ± 0.3 adults vs. 18.3 ± 1.7 nymphs). Adults that were in the middle of giving birth were quickly removed from the arena using gentle forceps and were not included in the collected data. In experiments where only nymphs were needed, 2–6 h before the beginning of the experiment all adult aphids were removed from the plant using forceps. When only adults were needed, they were placed on the stem 2–6 h before the experiment. This minimized the number of nymphs that dropped to the arena and any nymphs that did drop were quickly removed.

### Description and analysis of aphid behavior

The description and analysis of the riding behavior was done in three experiments, according to our three main questions:

### Experiment 1: Does riding behavior differ from the natural tendency of surface-walking aphids to climb on objects they encounter?

In this experiment, we compared the time nymphs spent on three types of objects: plastic beads (as schematic aphid models), dead adults and live adults. The experiment was necessary for determining whether the riding behavior is a new behavior or simply a manifestation of the tendency to climb on objects.

#### Plastic beads

The arena was constructed by arranging 64 thumbtacks with green, spherical, plastic bead heads (head diameter: 4 mm) that were tacked on a sheet of brown cardboard, in an 8X8 matrix (distance between adjacent insertion points was 1 cm). Only nymphs were dropped onto this arena. The arena was video filmed with a handheld Sanyo Xacti HD1010 high-definition video camera for 8 min in each trial. We used a video camera in this study since it provided detailed information on aphid movement and precise timing of events. We extracted data from the films on the movements of 35 individual nymphs (nymphs that did not contact other nymphs). We timed only the first climbing event of each nymph, from the moment it started climbing a bead until the moment it got off.

#### Dead adults

We constructed another arena, similar to the one described above, using dead adult aphids instead of thumbtacks. Sixty-four adult aphids were killed by freezing in -20 °C. The aphids were then arranged in an 8X8 matrix using fine forceps. The dead aphids were laid down facing forward and their limbs and antennae were arranged so that they were in a natural standing posture. Whenever an aphid’s color started turning dark (this happened with some of the aphids after several trials), it was replaced with a freshly killed aphid. The experiment was conducted and videotaped as described in the plastic bead experiment above (*n* = 35).

#### Live adults

This experiment was performed on a brown Bristol paper- covered table (table dimensions 2 X 1.5 m). Thirty created aphid colonies containing both adults and nymphs were separately used in this experiment (*n* = 30). Aphids were dislodged in the center of the arena and two observers manually timed riding events and clasified each adult as “static” or “mobile”. A static adult was defined as an adult that did not change its location while the nymph being tracked was on its body, as opposed to a mobile adult, which was defined as one that did change its location during that time. The observers timed each riding event from initial contact until the nymph was completely off the adult. Riding events of multiple nymphs on one adult were rare, and were not included in the collected data. In addition, the observers recorded whether and how the adults tried to remove the riding nymphs.

### Experiment 2: What are the costs and benefits of riding behavior?

To answer this question, we measured: a) the difference in movement speed between nymphs and adults. This data was a baseline for the following experiments; b) the effect of riding on return time to the plant. Shorter return times would indicate a benefit while longer return times would indicate a cost; c) the effect of riding on the chances of finding a plant. Better chances would indicate a benefit while worse chances would indicate a cost.

#### Difference in movement speed between nymphs and adults

A doughnut shaped trench (outer diameter: 28 cm; inner diameter: 5 cm; depth: 3 cm) was made in a Styrofoam board. The central circle that was left intact was covered with brown Bristol paper. This circle served as the starting point for the aphids that were dropped using a plastic funnel (described below). The trench was filled with dry commercial planting soil, which contained small pebbles and some small dry twigs. The surface of the soil was flat and flush with the middle circle and the trench’s edge. Thirty colonies were used in this experiment (*n* = 30). After the aphids were dropped onto the central circle, a stopwatch was started and the two observers recorded the time it took each aphid to reach the edge of the arena (the outer edge of the trench).

#### The effect of riding on return time and chances of finding a plant

This experiment was performed on a circular arena, surrounded by groups of lentil plants (details in Additional file 3). The high-definition video camera was positioned above the arena. The observers added information to the recorded video by providing verbal descriptions of events and using skewers to point out and refer to specific aphids.

At the beginning of each experiment, a plastic funnel (bottom/top opening diameters: 4/10 cm, respectively) was placed on the center of the arena and the aphids were dropped (*n* = 30 colonies) through the funnel, which was then removed. The funnel reduced the bouncing and scattering of aphids, thus raising the frequency of riding events and lowering the necessary number of trials. Although this caused some aphids to become temporarily entangled with each other, the aphids quickly disentangled. Tracking of aphid behavior always started after aphids disentangled.

To measure the probability of an aphid reaching a plant, we noted whether each aphid exited the arena in front of a plant or a gap. “Plant” was defined as a segment of the circumference of the arena that was planted with lentil plants and “gap” was defined as a segment that contained no plants. Only riding events that lasted until the adult exited the arena were included in the data, thus excluding partial and short riding events. In addition, during the experiments in the plant-surrounded arena we recorded the riding positions of nymphs on adults’ bodies.

### Experiment 3: do adults prefer carrying nymphs of their own kin?

In order to test whether adults prefer carrying nymphs of their own kin (an indication for parental care), we compared the riding frequency among members of our laboratory line of aphids (the one used in all other experiments in this study) with that of aphids from a mixed- line colony, where the chances of aphids encountering their kin are low. The mixed-line colony was established by 10 adult wingless pea aphids that were collected from 10 different locations near Haifa and Kiryat Tiv’on, northern Israel. The arena used in this experiment was a 20 cm diameter brown Bristol paper circle, pasted on a Styrofoam board. We performed two identical experiments, one with our laboratory line and one with the mixed-line colony. In each experiment we used 20 colonies (*n* = 20). The time it took each aphid to reach the edge of the arena was recorded. Only riding events that lasted until the adult exited the arena were recorded.

### Data analysis

It was not possible to record data blindly because our study involved close observation of the behavior of animals in the laboratory. All data were tested for normality using the Shapiro-Wilk test. Data that did not meet the assumptions of parametric tests were tested with equivalent nonparametric tests. All tests used were two-tailed. Specifications of the tests used are given in the results. Statistical analyses were performed using IBM SPSS software v.20. Means are presented with standard error in parentheses.

## Results

After dropping, adult aphids typically landed on their ventral side [3] and those that did not, quickly assumed an upright standing position. Nymphs typically started walking immediately after reaching the surface. We did not observe any long-distance attraction of nymphs to adults. However, nymphs that landed or passed at a distance of less than 5 mm from an adult, oriented toward the adult. When nymphs initiated a climbing attempt, the adults remained motionless or raised their bodies from the surface by extending their legs, thus making it difficult for the nymphs to climb on. In some cases, adults avoided nymphs by starting to walk once touched by a nymph. When reaching an adult, nymphs always tried to climb on the first body part they encountered and if they were on legs or antennae, they always continued moving until they reached the body.

Shortly after adults with riding nymphs reached a host plant and started feeding again, the nymphs came off and began feeding as well. On average, nymphs disembarked on the plant 3:11 ± 1:18 min (*n* = 15) after the adults started climbing the stem.

**Fig. 2:**
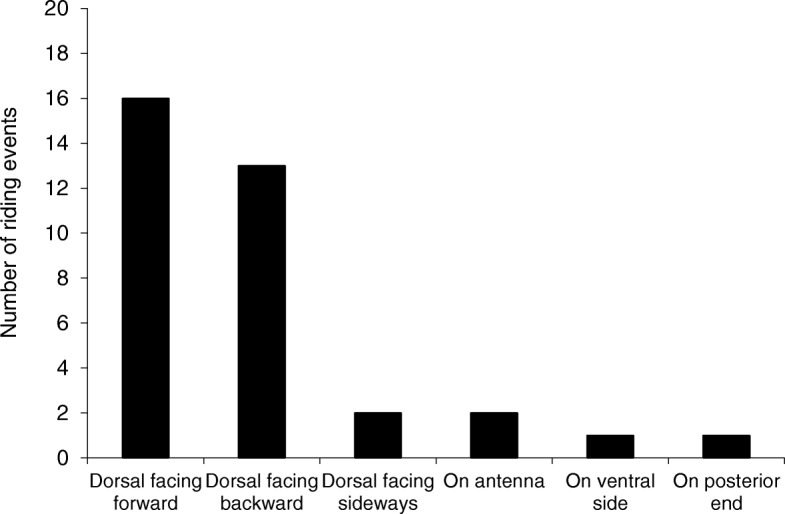
Riding positions of pea aphid nymphs on adults. The data refers to the riding of a single nymph on each adult. Dorsal facing forward: the riding position seen in Fig. 1; Dorsal facing backward / sideways: same as the previous position, with the nymph facing backwards or sideways, respectively. On ventral side: on any part of the adult’s body which is not the dorsum or the posterior end

The most frequent riding position was when the nymph was on the front dorsal part of the adult’s body, facing forward (*n* = 35, Figs. 1, 2). Nymphs usually stopped moving about after assuming this position and these riding events typically were the longest lasting ones.

Mobile adults often made active attempts to remove riding nymphs: 33.3% of the mobile adults that were carrying a nymph removed it from their anterior end by stopping and repeatedly lowering their head to the surface; 26.7% removed a nymph from their posterior end by stopping and repeatedly lowering it to the surface; 13.3% removed a nymph after performing both types of removal attempts. When nymphs were riding on the front dorsal part of the adult’s body facing forward (Fig. 1), adults made the smallest number of removal attempts and when they did try to remove the nymphs, they had the least success (data not shown).

### Experiment 1

**Fig. 3:**
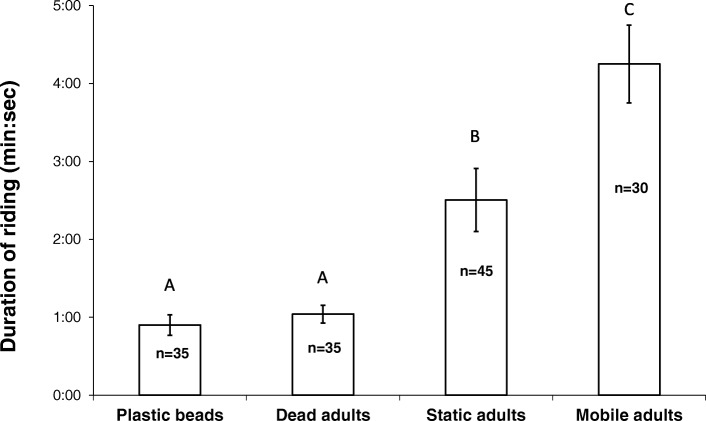
Duration of riding events of pea aphid nymphs on different objects. First instar nymphs were dropped onto an arena with small plastic beads, an arena with dead adult aphids and a large arena with live adults. The live adults were tracked until there were no more nymphs on them. Different letters above bars indicate significant differences

The amount of time nymphs stayed on an object they had climbed on depended on its nature. Nymphs spent less time on inanimate objects (plastic beads and dead adults) compared with the time they spent on live adults. When the live adults were motionless, the nymphs did not stay as long as they did when the adults were moving on the surface (Fig. 3; one way ANOVA, F_3,141_ = 28.934, R^2^ = 0.368, *P* < 0.001, post hoc Bonferroni-corrected t-test. Data was ln-transformed prior to analysis).

### Experiment 2

Adults not carrying nymphs exited the soil-covered arena after 4:14 ± 0:19 min (*n* = 102, including the time they spent standing at the landing site; adults that did not start walking after 10 min were excluded). The fastest 10 adults (ones that started walking almost immediately after dropping) exited the arena after 0:47 ± 0:04 min. Nymphs exited the arena after 25:15 ± 0:51 min (*n* = 167; nymphs did not stay at the landing site after dropping and therefore no nymphs were excluded from the analysis). The 10 fastest nymphs (ones that walked in a relatively straight line) exited after 8:55 ± 0:30 min. The average time it took adults to exit the arena was six times shorter than that of nymphs (Mann–Whitney U-test: U = 135.5, Z = − 13.6, *P* < 0.001) and the 10 fastest adults were 11.4 times faster than the 10 fastest nymphs (T – test: t_18_ = 16.2, *P* < 0.001).

In the plant-surrounded arena, the chances of nymphs reaching a plant were not affected by riding [66.9% for nymphs walking on their own (*n* = 438) vs. 65.9% for nymphs riding on adults (*n* = 41); Pearson’s Χ^2^_1_ = 0.18, *p* = 0.892]. However, riding on adults significantly shortened the time it took nymphs to exit the arena [8:06 ± 0:12 min for nymphs walking on their own (*n* = 438) vs. 1:55 ± 0:22 min for nymphs riding on adults (*n* = 17); T test: t_453_ = 8.7, *P* < 0.001]. Only cases of an adult carrying one nymph (the most common riding event) when it exited the arena were included in both analyses.

**Fig. 4:**
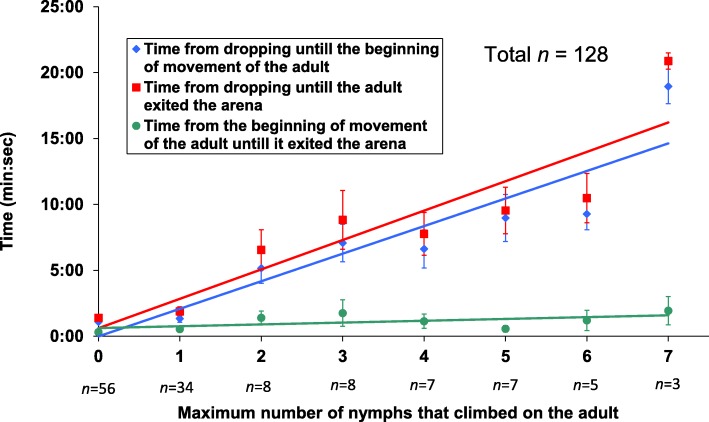
The effect of riding on adult movement: X axis denotes the highest number of nymphs that were observed on the adult’s body simultaneously, throughout the time it was in the arena. For each highest number of nymphs there was a different number of adults that were included in that category (numbers given underneath the X axis)

Without nymphs climbing on them, adults started walking 1:05 ± 0:07 min after dropping (*n* = 56). The duration of movement of an adult that was carrying one nymph, from the moment it started walking until it exited the arena, was similar to that of an adult not carrying nymphs (Mann–Whitney U-test: U = 551.5, Z = − 1.524, *P* = 0.127; Fig. 4). However, the higher the number of nymphs that were on it simultaneously, the longer it took to start moving after it dropped (Spearman’s rho = 0.596, *P* < 0.001; Fig. 4) and the longer the total time from dropping until it exited the arena (Spearman’s rho = 0.603, *P* < 0.001; Fig. 4). This was a result of the adults’ tendency of waiting until most nymphs came off their body before starting to walk. The duration of the walking itself was only slightly affected by the maximum number of nymphs climbing on the adult simultaneously (Spearman’s rho = 0.332, *P* < 0.001; Fig. 4).

### Experiment 3

Under our experimental conditions, 15% ± 2.8% of adults in the laboratory line exited the arena while carrying one or more nymphs (mean 1.1 ± 0.1) and 6.7% ± 1.2% of nymphs exited while riding on adults. Aphids from the mixed-line colony showed a similar riding frequency, as 15.9% ± 3.1% of adults exited the arena while carrying one or more nymphs, and 5% ± 1.3% of the nymphs exited while riding on adults. Riding frequencies of the mixed-line were not different from those of our laboratory line, for both adults (t-test, t_38_ = − 0.21, *p* = 0.835) and nymphs (Mann-Whitney U test, U = 154, *p* = 0.207).

## Discussion

Riding on the backs of adults after escape dropping lowers the exposure of young pea aphid nymphs to the hostile conditions and risk of starvation on the ground. By taking advantage of the agility of the adults, the nymphs compensate for their inferior mobility and low resistance to harsh environmental conditions [5, 7, 18–21].

The phenomenon of an animal “hitchhiking” on the body of another animal, thus dispersing from an area unsuitable for further development to a more favorable environment is referred to as phoresy [22]. Phoresy is usually an interspecific interaction, mostly described in arthropods and fish (e.g. [23–25]). Phoresy among members of the same species is rare and limited to males actively carrying females [26, 27]. The intraspecific phoresy in pea aphids presented here, where the hitchhiker initiates and the carrier is uninterested in carrying, has never been described before.

After dropping to the surface, the speed at which adult pea aphids start walking typically ranges from seconds to hours [28]. The adaptive significance of staying in one place after dropping to the ground, despite some recent works on aphid movement on horizontal surfaces [28, 29], is still unclear. Some aphids exhibit thanatosis after dropping (feigning death by retracting legs and antennae and remaining motionless) [14, 30], but in our system most adults quickly assumed an upright, alert standing posture (extended limbs and antennae) and remained at the landing site for about 1 min. (Fig. 4). The fact that nymphs started moving almost instantaneously while the adults stayed at their landing sites, which is similar to the findings of Roitberg et al. [14], increased their probability of climbing on adults, thus facilitating the riding behavior.

We found that nymphs stay longer on live adults (especially on moving ones) compared to inanimate objects (Fig. 3). This proves that riding differs from the aphids’ natural tendency to climb on objects (negative geotaxis) and is therefore a distinct behavior. On a moving adult, the nymphs apparently sense the movement and therefore stay for a longer time. The greater the difference of the object they climbed on from a moving adult, the faster they will disembark. The average riding time on live adults, that lasted about 4 min in the large arena (Fig. 3), should improve the chances of nymphs reaching a host plant. Since adults can walk much faster than nymphs (see also [15]), a few minutes of riding may be very significant for a nymph. If the adult returns to the same plant or arrives at an adjacent plant, riding that lasts a few minutes might be sufficient for bringing the nymph to a suitable host. All nymphs in our experiment successfully disembarked on a host plant and resumed feeding shortly after the adult climbed up the stem. Even if a nymph disembarks before reaching a host plant, riding part of the way will bring it closer to the plant, thus increasing its chances of survival.

We found that the improved chances of reaching a host plant are due to the shortened travel time on the surface and not due to a better ability of the adults to detect host plants (Fig. 4). However, this conclusion is based on experiments conducted in a laboratory. Roitberg et al. [14] found that in the field, older instar pea aphids are better at locating new host plants than younger instars, a difference that would make the riding behavior even more beneficial for the nymphs.

For the adult, riding behavior may be costly. The climbing of several nymphs delays its departure from the landing site (Fig. 4) and the removal attempts may also slow it down. Delayed departure and removal attempts are expected to lengthen the time an adult aphid spends on the ground, thus increasing its exposure to danger. The fact that the adults actively try to remove the nymphs is an indication to the irritating nature and the disadvantage of the riding behavior for the adults. The preference of nymphs for riding on the front dorsal part of the adult (Figs. 1, 3) may be do to the lower chance of being removed.

Carrying offspring by adults is a common phenomenon throughout the animal kingdom, despite the high immediate cost for the carrying adult. For example, most primates and all scorpions carry their young [31, 32]. However, except for a few rare cases of parental defense in gall-forming aphids (e.g. [33]), there are no reports of parental care in aphids. Also, kin recognition is not found in aphids, although some degree of non-kin recognition ability was suggested among two biotypes of pea aphids [34]. We did not find evidence for parental care or kin recognition, as in the mixture of aphids from 10 different lines, where the probability of encounter between nymph and adult from the same line was low, riding frequency was similar to that of our laboratory monoclonal colony (experiment 3). If nymphs and adults in the mixed line colony would have sought or assisted members of their own line or specifically avoided non-kin, riding frequency would have been lower due to frequent mismatches. In addition, the fact that adults may actively prevent the nymphs from climbing and even try to remove them indicates that this behavior is not a type of parental care.

Rare laboratory observations of pea aphid nymphs climbing on adults have been attributed to “cannibalism” [35, 36], since the nymphs were sometimes seen contacting the adults’ bodies with their proboscis. We did observe riding nymphs extending their proboscis and touching the adults’ bodies (probing), but those events were rare and always lasted less than 2 min. The actual insertion of stylets into the adult’s body was observed once, when a stationary riding event was examined under the microscope. Since it was a rare and short-lived event, we speculate that it is part of the normal probing behavior of aphids [37], which will probe many surfaces including paper and glass [38].

The probability of riding events occurring after aphids escape from an insect predator or parasitoid is low, because dropping pattern in response to insect enemies is usually intermittent [8], as opposed to the concurrent instantaneous dropping that occurs in response to mammalian herbivory [9]. Also, when escaping from insect enemies, adults typically tend to drop significantly more than nymphs [18, 39, 40]. This difference between adults and nymphs is at least partially explained by differences in mobility and survival on the ground. However, in response to the combined stimulation of warm breath and plant vibrations which is typical of mammalian herbivory, first instar nymphs will also drop off the plant in large numbers [10]. Therefore, aphids are expected to demonstrate riding behavior more often after escaping from a foraging mammalian herbivore than during the intermittent dropping that characterizes the response to a foraging insect enemy.

Nevertheless, when an attack by an arthropod predator or even intense rain [41–43] dislodges a significant number of adults and young nymphs in a short period of time, riding may also take place.

When aphids flee from a foraging mammalian herbivore, young and old aphids enter a conflicting situation; for the nymph, climbing and riding on an adult will significantly reduce the time spent on the ground before reaching a plant. For the adult, riding nymphs are a nuisance. The fact that riding behavior exists indicates that it increases the inclusive fitness of the colony, i.e. the benefit for the nymphs outweighs the cost for the adults. Future research that will be conducted in different natural habitats will improve our understanding of this novel behavior and contribute to the multifaceted research of the direct interactions between mammalian herbivores and plant-dwelling insects.

## Conclusions

In the face of danger, clonal animals may benefit from assisting other (genetically identical) colony-members in need. We studied a novel “riding behavior” of wingless pea aphids that are challenged with the need of locating a host plant after dropping to the ground in response to danger. By observing and recording aphid behavior, we found that after dropping, young aphids quickly climb on adults and stay on their backs. The adults then walk back to a host plant, carrying one or more young. This assists the young in reaching a host plant, but delays the adults, which often actively try to remove the young. The frequency of occurrence of this behavior did not depend on the level of relatedness of the participating conspecifics. We therefore did not find evidence of adults assisting young colony members.

## Additional files

Additional file 1: Video of a first instar pea aphid riding on the back of an adult. (MOV 3336 kb)

Additional file 3: Plant-surrounded arena for surface-movement experiments with pea aphids. The arena was constructed from a 60 cm diameter and 5 cm thick circular Styrofoam board (A). A 30 cm diameter circle, cut out of a thin, brown, plastic sheet, was glued onto the center of the arena (B). A 28 cm diameter inner circle cut out of brown Bristol paper (C) was glued in the middle of the plastic circle, leaving a 1 cm band of plastic without paper, which prevented the paper from absorbing water from the wet soil that surrounded the arena (D). The wet soil surrounding the arena (commercial planting medium) was in a 10 cm wide and 3 cm deep circular trench that was dug into the Styrofoam. The circumference of the arena was divided into 12 segments. Lentil plants were planted in six of the segments on the edge of the arena (E), so that 50% of the circumference of the arena was lined with a dense “forest” of lentil plants. Each aphid that reached and touched the border between the plastic sheet and the Bristol paper (the border between B and C) was considered as exiting the arena at that point. (TIF 2097 kb)
