# Supplementary figures and images for: Standing on the shoulders of giants: young aphids piggyback on adults when searching for a host plant
Source: Front Zool. 2018 Dec 6;15:49. doi: 10.1186/s12983-018-0292-7 (PMC6282293; doi:10.1186/s12983-018-0292-7)

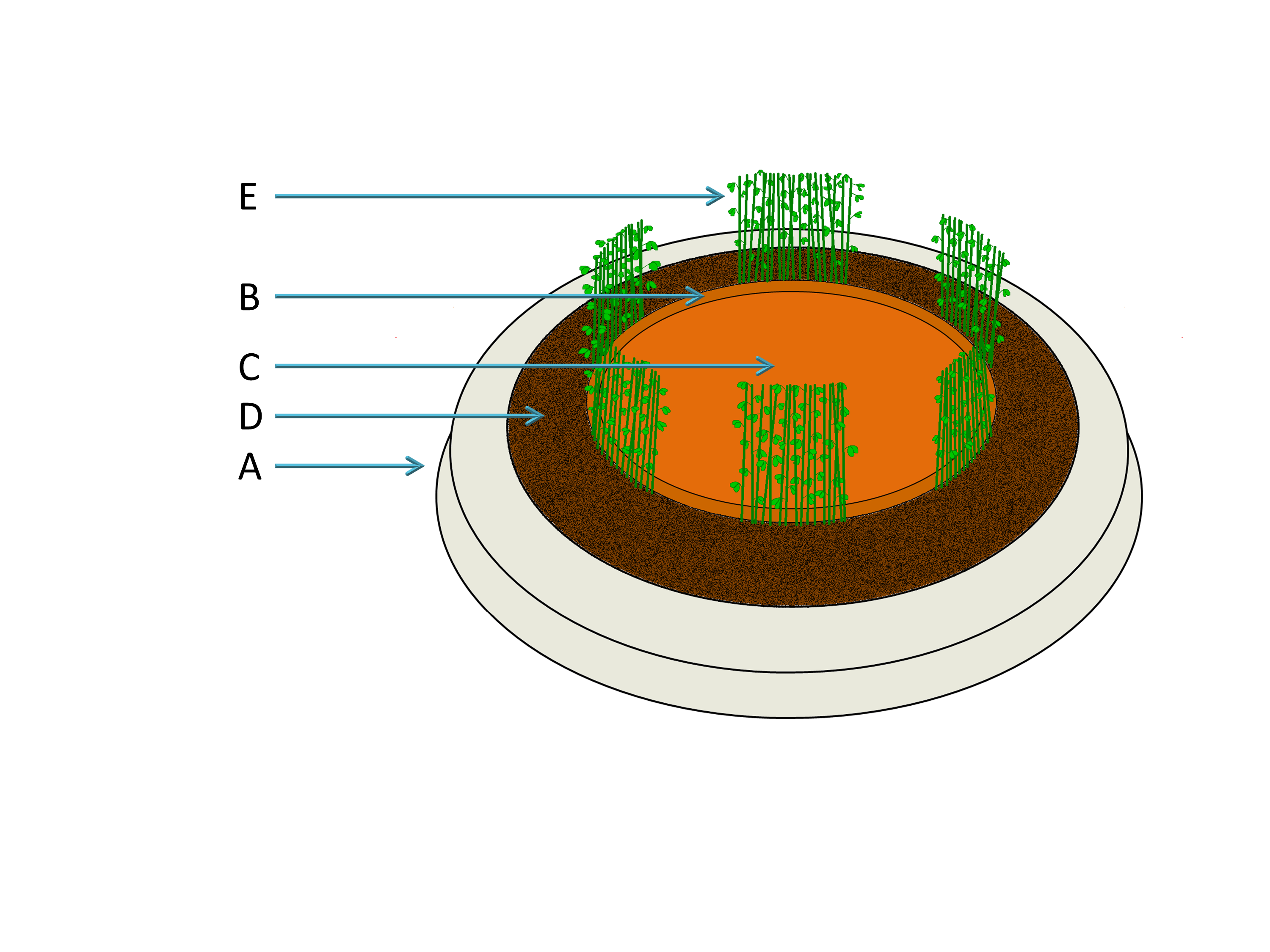

Supplement: Supplementary file 3 — Plant-surrounded arena for surface-movement experiments with pea aphids. The arena was constructed from a 60 cm diameter and 5 cm thick circular Styrofoam board (A). A 30 cm diameter circle, cut out of a thin, brown, plastic sheet, was glued onto the center of the arena (B). A 28 cm diameter inner circle cut out of brown Bristol paper (C) was glued in the middle of the plastic circle, leaving a 1 cm band of plastic without paper, which prevented the paper from absorbing water from the wet soil that surrounded the arena (D). The wet soil surrounding the arena (commercial planting medium) was in a 10 cm wide and 3 cm deep circular trench that was dug into the Styrofoam. The circumference of the arena was divided into 12 segments. Lentil plants were planted in six of the segments on the edge of the arena (E), so that 50% of the circumference of the arena was lined with a dense “forest” of lentil plants. Each aphid that reached and touched the border between the plastic sheet and the Bristol paper (the border between B and C) was considered as exiting the arena at that point. (TIF 2097 kb) [file 12983_2018_292_MOESM3_ESM.tif]
